# Gender differences in alcohol abuse/dependence among medical undergraduates during the post-COVID‑19 pandemic period (October 20, 2020-April 5, 2021) in China

**DOI:** 10.1186/s12888-023-05260-3

**Published:** 2023-10-16

**Authors:** Xin Wang, Pu Peng, Yueheng Liu, Winson Fuzun Yang, Shubao Chen, Yunfei Wang, Qian Yang, Manyun Li, Yingying Wang, Yuzhu Hao, Li He, Qianjin Wang, Junhong Zhang, Yuejiao Ma, Haoyu He, Yanan Zhou, Jiang Long, Chang Qi, Yi-Yuan Tang, Yanhui Liao, Jinsong Tang, Qiuxia Wu, Tieqiao Liu

**Affiliations:** 1 Department of Psychiatry, National Clinical Research Center for Mental Disorders, and National Center for Mental Disorders, The Second Xiangya Hospital of Central South University, Changsha, 410011 Hunan China; 2 Department of Psychological Sciences, Texas Tech University, Lubbock, TX USA; 3 School of Physical Education and Health, Hunan University of Technology and Business, Changsha, China; 4 Department of Psychology, College of Education, Hunan First Normal University, Changsha, China; 5 Department of Psychiatry, Hunan Brain Hospital (Hunan Second People’s Hospital, Changsha, China; 6 Shanghai Mental Health Center, Shanghai Jiao Tong University School of Medicine, Shanghai, China; 7 Department of Psychiatry, Zhejiang Provincial People’s Hospital, People’s Hospital of Hangzhou Medical College, Hangzhou, Zhejiang P. R. China; 8 College of Health Solutions, Arizona State University, Phoenix, AZ USA; 9 Department of Psychiatry, Sir Run Shaw Hospital, School of Medicine, Zhejiang University, Hangzhou, Zhejiang P. R. China

**Keywords:** Alcohol abuse/dependence, Learning burnout, Anxiety, Gender differences, The coronavirus disease 2019

## Abstract

**Background:**

This study aimed to assess the prevalence and the gender-specific risk factors of alcohol abuse/dependence among medical undergraduates during the post-COVID‑19 pandemic period in China.

**Method:**

The Alcohol Use Disorders Identification Test-Consumption (AUDIT-C) was used to identify respondents with alcohol abuse/dependence. A questionnaire on basic demographics and mental distresses (learning burnout, depression symptoms, anxiety symptoms, excessive daytime sleepiness, and history of mental disorders) was used. The logistic regression model was used to explore the associations between the above characteristics and alcohol abuse/dependence.

**Results:**

A total of 3,412 medical undergraduates were included in the analysis. Males showed a higher prevalence of alcohol abuse/dependence than females (16.6% vs 7.4%, *p* < 0.001). Alcohol abuse/dependence was associated with learning burnout (OR: 2.168, *p* < 0.001) and having a partner (OR: 1.788 *p* = 0.001) among female medical undergraduates. Among male medical undergraduates, excessive daytime sleepiness (OR: 1.788 *p* = 0.001) and older age (OR: 1.788, *p* = 0.001) were independently associated with alcohol abuse/dependence.

**Conclusion:**

Alcohol abuse/dependence was common among medical undergraduates during the post-COVID‑19 pandemic period. Substantial gender differences in the prevalence and risk factors of alcohol abuse/dependence were found among medical undergraduates in this study, which highlighted the need for timely gender-specific screening and interventions. However, the cross-sectional design adopted in this study has limited the examination of causality, thus further longitudinal studies are warranted.

## Introduction

Alcohol abuse/dependence is defined as a maladaptive pattern of drinking that results in clinically significant impairment or distress [1]. Recently, alcohol abuse/dependence among medical students is a serious public health concern. Multiple studies have demonstrated that alcohol is one of the most frequently used substances among medical students [2, 3]. Many medical students reported alcohol-related disorders, ranging from 32.4 to 34.4% in the United States [4, 5], 19.2% in Norway [6], 22.6% in the Republic of Korea [7], 21% in France [8] and 4.57 to 8.25% in China [9, 10].

Previous studies have suggested that alcohol abuse/dependence among medical students can have serious personal and public ramifications. On the one hand, alcohol abuse/dependence in medical students is associated with an increased risk of physical and mental health problems, such as cirrhosis, pancreatitis, and suicide behavior [11, 12]. On the other hand, alcohol abuse/dependence is often accompanied by antisocial behaviors, alcohol impaired driving, and violence, which can have negative impacts on the whole society [13, 14]. In addition, there may be potential long-term consequences of alcohol abuse/dependence in medical students. Previous studies suggested that alcohol use during medical training significantly increases the risk of alcohol use disorders later in their careers as physicians [15–18], which increases the risk of medical errors and contributes partly to the disturbingly high divorce and suicide rates [19, 20]. Moreover, it is also worth noting that drinking alcohol at any age is legal in China, which means that medical students are not legally restricted in their purchase and use of alcohol. Therefore, it is necessary to early identify alcohol abuse/dependence among medical students, in order to reduce their risk of short- and long-term adverse consequences.

A number of researchers have explored related factors associated with alcohol abuse/dependence. Firstly, a growing body of evidence has demonstrated gender differences in a variety of aspects of alcohol use in the general population. For example, males are more likely than females to engage in harmful drinking [21–24]. Besides, the predictive effects of many psycho-sociocultural variables (such as anxiety disorders) on alcohol-related problems also differ between males and females [25, 26]. Gender differences in physiological (e.g., alcohol pharmacokinetics and the effects of alcohol on hormones) and socio-cultural factors may account for these differences [23]. Secondly, previous researches had indicated that alcohol abuse/dependence may be associated with mental distresses [5, 27, 28]. For example, a study of 4,402 US medical students suggested that alcohol abuse/dependence was strongly related to burnout [5]. In addition, depression, anxiety, and sleep-related problems might also be related factors to alcohol abuse/dependence [29–32]. Notably, the outbreak of coronavirus disease 2019 (COVID-19) may also have had a negative impact on alcohol relative problems [33]. An increase in alcohol consumption was observed among college students [34, 35] and general population [36] during the COVID-19 pandemic. Furthermore, researchers suggested that the negative mental health consequences caused by the measures to curb COVID-19 transmission might be long-lasting even after the public health risk has been abated [37]. Therefore, it is important to screen medical students for alcohol abuse/dependence even after the pandemic.

However, some gaps in current research on alcohol abuse/dependence among medical students exist. Firstly, gender differences regarding alcohol abuse/dependence among medical students have been inconsistent across studies. Some studies reported no difference in the percentages of at-risk drinkers between male and female medical students [38, 39], while a study conducted in Spain indicated that female medical students were more likely to be “at risk” [40]. Secondly, medical school is a significant period of psychological distress for students [41, 42]. Mental distress such as burnout, depression, and anxiety are prevalent among medical students [43]. However, no current research has explored whether mental distresses have different effects on alcohol abuse/dependence between different genders. Lastly, previous studies of alcohol abuse/dependence among medical students during the COVID-19 pandemic involved only a small number of participants [44–46], and little is known about this problem among Chinese medical students after the outbreak of COVID-19. Therefore, gender differences in the prevalence and correlations of alcohol-related disorders among medical students during the post-COVID‑19 pandemic period are worthy of further investigation.

This study aimed to identify the prevalence and gender-specific related factors for alcohol abuse/dependence among Chinese medical undergraduates during the post-COVID‑19 pandemic period. We hypothesized that 1) alcohol abuse/dependence is common among Chinese medical undergraduates during the post-COVID‑19 pandemic period, 2) compared to female medical undergraduates, male students are more likely to engage in alcohol abuse/dependence, and 3) among Chinese medical undergraduates, the association between alcohol abuse/dependence and mental distresses (i.e., learning burnout, depression symptoms, anxiety symptoms, excessive daytime sleepiness, and history of mental disorders) may significantly differ between males and females.

## Method

### Study design and participants

A cross-sectional web-based survey was conducted between October 20, 2020, and April 5, 2021 in China. During this period, the COVID-19 pandemic was subsiding in most areas of China and medical undergraduates were returning to campus. This study is a part of a large online study on the mental health of medical students and professionals in China [47, 48]. We used the snowballing sampling technique to recruit participants. The inclusion criteria included 1). undergraduate medical students; 2). Age greater than or equal to 18 years old. The excluded criteria were as follows:1). non-completion of the questionnaire; 2). invalid questionnaires. The researcher predetermined the criteria for determining an invalid questionnaire: (1) wrong answers for the general knowledge question; (2) all the answers are the same throughout the questionnaire; and (3) there is inconsistency in the logical consistency check.

### Sample size

A sample size of 3008 produces a two-sided 95% confidence interval with a width equal to 0.020 when the sample proportion is 0.083. Sample proportion based on alcohol abuse/dependence reported by 8.25% of Chinese medical students in previous study [9]. The sample size was calculated using PASS 15.0.5. Given the possibility of invalid questionnaires, the sample size should be increased by 10%. Therefore, this study requires at least 3,308 participants.

### Data collection

In order to recruit a large number of participants as conveniently as possible, we used the snowballing sampling technique. The data were collected via an online survey platform (http://www.wjx.cn) and a social media platform (WeChat) in China. First, 40 medical students and medical university teachers were selected as the “original delivers”, all of whom were members of this research group. They shared this survey link through WeChat group and Moments to invite their classmates or students to participate in this survey. These delivers and potential participants are WeChat friends or in the same WeChat group. By clicking on the survey link to participate in the study. In addition, participants were also encouraged to share this survey link via their WeChat group and Moments. Only participants who responded to all the questions were able to submit the questionnaire, thus, there were no missing data in this study.

### Questionnaire development

The online questionnaire was written in Chinese. This survey included items on alcohol abuse/dependence, mental distresses (learning burnout, depression symptoms, anxiety symptoms, excessive daytime sleepiness, and history of mental disorders), as well as basic demographic information (age, gender, relationship status, monthly income, and years of school). Before starting the final questionnaire dissemination, it was piloted on 20 medical students. These piloted medical students were not included in the final analysis.

#### Alcohol abuse/dependence

The Alcohol Use Disorders Identification Test-Consumption (AUDIT-C) was used to identify respondents with alcohol abuse/dependence [49]. The Chinese version of AUDIT-C has been tested for validity and reliability [50]. The AUDIT-C consists of 3 items, assessing the amount and frequency of alcohol consumption as well as the frequency of having 6 or more standard drinks on one occasion. The total score of AUDIT-C ranges from 0 to 12, with a cut-off point of ≥ 3 (for female students) or ≥ 4 (for male students) indicating alcohol abuse/dependence [51]. The AUDIT-C is highly reliable with reliability coefficient (Cronbach's α) of 0.828.

#### Learning burnout

The Learning Burnout of Undergraduates Scale (LBUS) was used to identify respondents with learning burnout [52]. This scale was developed specifically to assess learning burnout among college students and has been widely used in China [53]. The LBUS includes 20 items, with a total score ranging from 20 to 100 and a cut-off point of 60 indicating learning burnout [54]. LBUS has strong internal reliability with Cronbach's α of 0.898.

#### Depression and anxiety

The Chinese version of the 9-item Patient Health Questionnaire (PHQ-9) and the 7-item Generalized Anxiety Disorder Scale (GAD-7) were used to assess the respondents’ depression and anxiety symptoms [55, 56], with a cut-off point of 10 in PHQ-9 indicating the presence of moderate or severe depression symptoms [57] and a cut-off point of 10 in GAD-7 indicating the presence of moderate or severe anxiety symptoms [58]. The reliability coefficients (Cronbach's α) for the GAD-7 and PHQ-9 showed excellent internal consistency, which was 0.908 and 0.871 respectively.

#### Excessive daytime sleepiness

The Epworth Sleepiness Scale (ESS) was used to assess daytime sleepiness among participants [59]. The Chinese version of ESS is a reliable and widely used questionnaire in China [60]. Participants with a total score of ≥ 11 were considered to have excessive daytime sleepiness [61]. The reliability of ESS is acceptable with Cronbach's α of 0.771.

### Data quality control and bias

Firstly, each IP address can only fill in the questionnaire once to avoid repeated data collection. Secondly, the criteria for invalid questionnaires were set in advance by the researcher and the sample size was higher than the minimum required sample size. Therefore, the effect of deleting the invalid questionnaire on the results of the study was negligible.

### Statistical analysis

We used standard descriptive statistics to characterize the responding medical undergraduates. To screen out the independent variables associated with alcohol abuse/dependence in the male and female groups, we first performed univariate analysis by using chi-square test and Mann–Whitney U test, as appropriate, to compare inter-group differences in related variables. Variables with significant inter-group differences in the univariate analysis (*p* < 0.05) were further included in a multivariable logistic regression model. The independent variables used in the two-step screening included mental distresses (learning burnout, depression symptoms, anxiety symptoms, excessive daytime sleepiness, and history of mental disorders) and basic demographic data (age, gender, relationship status, monthly income, and years of school). All the statistical analyses were performed using SPSS Version 26 (IBM, Armonk, NY, USA). All tests were 2-tailed with a significance level of 0.05.

### Ethical considerations

This study was approved by the ethics committee of the Second Xiangya Hospital of Central South University (LYF2020075). All the participants were voluntary, with informed consent provided through an online survey platform (http://www.wjx.cn). Immediately after completing the survey, participants will receive a report on their mental health status and recommendations to promote their mental health status. In addition, participants will be given the contact information of our team of mental health professionals to enable them to contact us for help.

## Result

### participants’ characteristics

**Fig. 1:**
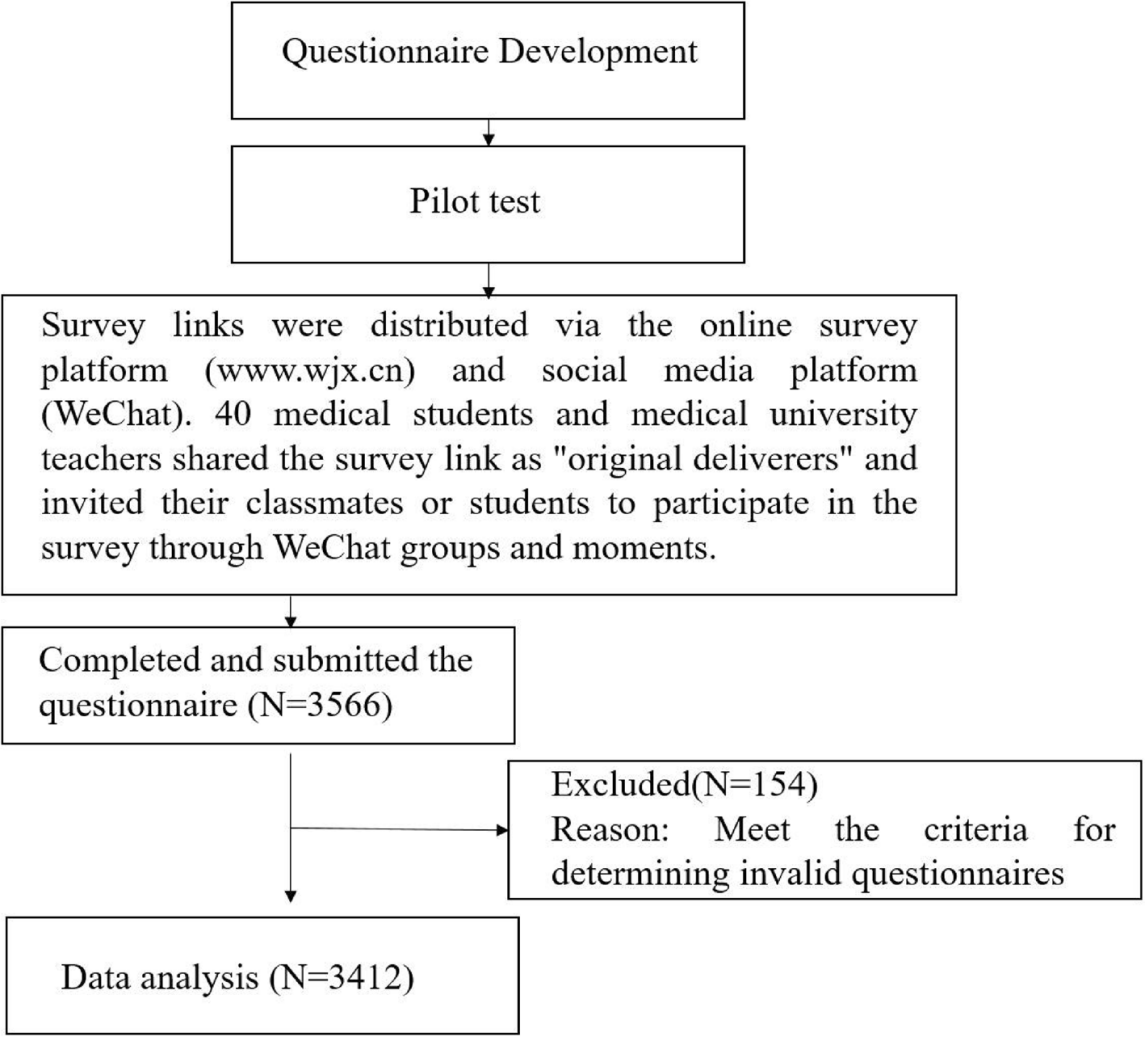
Flowchart of the recruitment process

A total of 3,566 medical students participated in this online survey. Through data screening, 154 respondents were deemed invalid and excluded from the analysis. Of the 154 excluded participants, 32 had incorrect answers to the general knowledge questions and 41 had the same answers throughout the questionnaire; 81 participants had inconsistencies in the logical consistency check. Thus, 3,412 participants were included in the final analysis, with the efficiency rate of 95.7%. The flow chart for participant recruitment is shown in Fig. 1. The participant characteristics (overall and by gender) are listed in Table 1. In this study, there were 1067 (31.3%) male and 1,027 (68.7%) female. Their age range was mainly 18–20 years (72.3%), relationship status was mainly single (78.4%), year in school range mainly 1–3 years (87%), and monthly income was mainly < 615 CNY (47.7%). In the whole studied population, a total of 350 (10.3%) medical undergraduates met the criteria for alcohol abuse/dependence, and the prevalence of learning burnout, depression symptoms, anxiety symptoms, excessive daytime sleep, having a history of mental disorders was 38.3%, 17.9%, 8.9%, 42.9%, and 2.9%, respectively.

**Table 1:** Characteristics of participants

Characteristics	Total	Male	Female	χ2/Z	*P* value
	*N* = 3412	*N* = 1067	*N* = 2345		
Age				-1.16	0.25
18–20	2468 (72.3%)	788 (73.9%)	1680 (71.6%)		
21–23	913 (26.8%)	262 (24.6%)	651 (27.8%)		
> 23	31 (0.9%)	17 (1.6%)	14 (0.6%)		
Relationship status				3.71	0.05
Single	2675 (78.4%)	858 (80.4%)	1817 (77.5%)		
Partnered	737 (21.6%)	209 (19.6%)	528 (22.5%)		
Year of school				-4.35	< 0.001
1	879 (25.8%)	369 (34.6%)	510 (21.7%)		
2	968 (28.4%)	249 (23.3%)	719 (30.7%)		
3	1122 (32.9%)	289 (27.1%)	833 (35.5%)		
4	310 (9.1%)	108 (10.1%)	202 (8.6%)		
5	133 (3.9%)	52 (4.9%)	81 (3.5%)		
Monthly income				-4.29	< 0.001
< 615 CNY	1626 (47.7%)	471 (44.1%)	1155 (49.3%)		
616–1310 CNY	1048 (30.7%)	299 (28.0%)	749 (31.9%)		
1311–2086 CNY	617 (18.1%)	257 (24.1%)	360 (15.4%)		
> 2086 CNY	121 (3.5%)	40 (3.7%)	81 (3.5%)		
Alcohol abuse/dependence	350 (10.3%)	177 (16.6%)	173 (7.4%)	67.59	< 0.001
Learning burnout	1308 (38.3%)	436 (40.9%)	872 (37.2%)	4.19	0.041
Depression	610 (17.9%)	183 (17.2%)	427 (18.2%)	0.56	0.46
Anxiety	304 (8.9%)	86 (8.1%)	218 (9.3%)	1.381	0.24
Excessive daytime sleepiness	1464 (42.9%)	359 (33.6%)	1105 (47.1%)	54.36	< 0.001
History of mental diseases	100 (2.9%)	28 (2.6%)	72 (3.1%)	0.51	0.47

### Comparison of alcohol abuse/dependence, mental distresses, and demographic characteristics between male and female medical students

Compared with females, male students showed a higher prevalence of alcohol abuse/dependence (16.6% vs 7.4%, *p* < 0.001). This difference remained significant after controlling for characteristics including age, relationship status, monthly income, anxiety symptoms, depression symptoms, learning burnout, excessive daytime sleepiness, and history of mental disorders (B = 1.009, Wald statistic = 68.804, *p* < 0.001, odds ratio [OR]: 2.742, 95% confidence interval [95%CI]: 2.161–3.481). In addition, the male students showed a higher prevalence of learning burnout (40.9% vs 37.2%, *p* = 0.041) and a lower prevalence of excessive daytime sleepiness (33.6% vs 47.1%, *p* < 0.001) than their female counterparts. Gender differences in the proportion of years of school, and monthly income were also significant (both *p* < 0.001, Table 1). There were no significant differences in the proportion of age, relationship status, depression symptoms, anxiety symptoms, or history of mental disorders between female and male students (all *p* > 0.05, Table 1).

### Differences in the comparison of mental distresses and demographic characteristics between male medical students with and without alcohol abuse/dependence

The univariate analyses of male medical students with alcohol abuse/dependence demonstrated that alcohol abuse/dependence was associated with learning burnout, depression symptoms, anxiety symptoms, excessive daytime sleepiness and a history of mental disorders (all *p* < 0.05, Table 2). In addition, inter-group differences were also significant in the proportion of age, relationship status, and monthly income (all *p* < 0.05). We then performed a multivariate logistic regression and variables with *p* < 0.05 in the univariate analysis were included in this model (Table 3). The results of the Hosmer–Lemeshow goodness-of-fit test showed that χ2 = 5.816, *P* = 0.561, indicating that the model fit was good. Multivariate logistic regression indicated that alcohol abuse/dependence was significantly associated with excessive daytime sleepiness (OR: 2.133, 95%CI: 1.508–3.018, *p* < 0.001), anxiety symptoms (OR: 2.766, 95%CI: 1.668–4.587, *p* < 0.001), and a history of mental disorders (OR: 2.527, 95%CI: 1.084–5.887, *p* = 0.032). Demographic variables independently associated with alcohol abuse/dependence included the age of 21–23 years (vs. 18–20 years, OR: 1.672, 95%CI: 1.155–2.422, *p* = 0.007) and higher monthly income (1,311–2,086 Chinese yuan [CNY] vs. under 615 CNY, OR: 2.367, 95%CI: 1.569–3.570, *P* < 0.001; > 2,086 CNY vs. 615 CNY, OR: 2.318, 95%CI: 1.048–5.127, *P* = 0.038).

**Table 2:** Characteristics of male and female medical undergraduates with and without alcohol abuse/dependence

Characteristics	Male		χ2/Z	Female		χ2/Z
	Without alcohol abuse/dependence	With alcohol abuse/dependence		Without alcohol abuse/dependence	With alcohol abuse/dependence	
	(*N* = 890)	(*N* = 177)		(*N* = 2172)	(*N* = 173)	
Age			-3.13^*^			-1.43
18–20	674 (75.7%)	114 (64.4%)		1564 (72.0%)	116 (67.1%)	
21–23	203 (22.8%)	59 (33.3%)		596 (27.4%)	55 (31.8%)	
> 23	13 (1.5%)	4 (2.3%)		12 (0.6%)	2 (1.2%)	
Relationship status			8.83^*^			17.39^*^
Single	730 (82.0%)	128 (72.3%)		1705 (78.5%)	112 (64.7%)	
Partnered	160 (18.0%)	49 (27.7%)		467 (21.5%)	61 (35.3%)	
Year of school			-1.45			-0.04
1	321 (36.1%)	48 (27.1%)		475 (21.9%)	35 (20.2%)	
2	196 (22.0%)	53 (29.9%)		656 (30.2%)	63 (36.4%)	
3	241 (27.1%)	48 (27.1%)		786 (36.2%)	47 (27.2%)	
4	94 (10.6%)	14 (7.9%)		185 (8.5%)	17 (9.8%)	
5	38 (4.3%)	14 (7.9%)		70(3.2%)	11 (6.4%)	
Monthly income			-4.24^*^			-4.58^*^
< 615 CNY	412 (46.3%)	59 (33.3%)		1094 (50.4%)	61 (35.3%)	
616–1310 CNY	255 (28.7%)	44 (24.9%)		689 (31.7%)	60 (34.7%)	
1311–2086 CNY	194 (21.8%)	63 (35.6%)		324 (14.9%)	36 (20.8%)	
> 2086 CNY	29 (3.3%)	11 (6.2%)		65 (3.0%)	16 (9.2%)	
Learning burnout	345 (38.8%)	91 (51.4%)	9.77^*^	770 (25.5%)	102 (59.0%)	37.91^*^
Depression	127 (14.3%)	56 (30.4%)	31.35^*^	363 (16.7%)	64 (31.5%)	44.26^*^
Anxiety	52 (5.8%)	34 (14.3%)	35.60^*^	176 (8.1%)	42 (16.1%)	49.71^*^
Excessive daytime sleepiness	268 (30.1%)	91 (51.4%)	30.00^*^	1001 (46.1%)	104(60.1%)	12.66^*^
History of mental disorders	16 (1.8%)	12 (4.6%)	14.34^*^	54 (2.5%)	18 (10.4%)	33.76^*^

^*^: p < *0.05*

**Table 3:** Characteristics of male and female medical students with alcohol abuse/dependence (logistic regression model)

	Characteristics	B	Wald statistic	P	OR	95%CI
Male^a^	Anxiety	1.017	15.536	< 0.001	2.766	1.668–4.587
	History of mental diseases	0.927	4.612	0.032	2.527	1.084–5.887
	Excessive daytime sleepiness	0.757	18.310	< 0.001	2.133	1.508–3.018
	Age 21–23 (versus 18–20)	0.514	7.406	0.007	1.672	1.155–2.422
	1311–2086 CNY (versus < 615 CNY)	0.862	16.890	< 0.001	2.367	1.569–3.570
	> 2086 CNY (versus < 615 CNY)	0.841	4.308	0.038	2.318	1.048–5.127
Female^b^	Anxiety	0.909	18.809	< 0.001	2.481	1.645–3.741
	History of mental diseases	1.069	12.420	< 0.001	2.912	1.607–5.277
	Learning burnout	0.774	20.566	< 0.001	2.168	1.552–3.030
	Relationship status	0.581	11.137	0.001	1.788	1.271–2.516
	616–1310 CNY (versus < 615 CNY)	0.504	6.866	0.009	1.656	1.135–2.414
	1311–2086 CNY (versus < 615 CNY)	0.684	9.179	0.002	1.982	1.273–3.086
	> 2086 CNY (versus < 615 CNY)	1.211	13.341	< 0.001	3.357	1.753–6.430

^a^In the regression model of male, variables included demographic characteristics (age, relationship status, and monthly income) and mental distress (learning burnout, depression symptoms, anxiety symptoms, excessive daytime sleepiness, and history of mental disorders)

^b^In the regression model of female, Variables included demographic characteristics (relationship status and monthly income) and mental distress (learning burnout, depression symptoms, anxiety symptoms, excessive daytime sleepiness, and history of mental disorders)

### Differences in the comparison of mental distresses and demographic characteristics between female medical students with and without alcohol abuse/dependence

Female medical students with alcohol abuse/dependence in our study showed higher prevalence of learning burnout, depression symptoms, anxiety symptoms, excessive daytime sleepiness and a history of mental disorders, as compared with those without alcohol abuse/dependence (all *p* < 0.05, Table 2). Inter-group differences were also significant in the proportion of monthly income and relationship status (all *p* < 0.05). Then a multivariate logistic regression was performed (Table 3). The results of the Hosmer–Lemeshow goodness-of-fit test showed that χ2 = 0.949, *P* = 0.813, indicating that the model fit was good. Multivariate logistic regression indicated that alcohol abuse/dependence was associated with learning burnout (OR: 2.168, 95%CI: 1.552–3.030, *p* < 0.001), anxiety symptoms (OR: 2.481, 95%CI: 1.645–3.741, *p* < 0.001), and a history of mental disorders (OR: 2.912, 95%CI: 1.607–5.277, *p* < 0.001) (Table 3). Demographic variables independently associated with alcohol abuse/dependence included having a partner (vs. single, OR: 1.788, 95%CI: 1.271–2.516, *p* = 0.001), and a monthly income over 615 CNY (616–1,310 CNY vs. under 615 CNY, OR: 1.656, 95%CI: 1.135–2.414, *p* = 0.009; 1,311–2,086 CNY vs. under 615 CNY, OR: 1.982, 95%CI: 1.273–3.086, *p* = 0.002; > 2,086 CNY vs. 615 CNY, OR: 3.357, 95%CI: 1.753–6.430, *p* < 0.001).

## Discussion

### Main findings

To our knowledge, this is the first study on the prevalence and gender-specific risk factors of alcohol abuse/dependence among Chinese medical students during the post-COVID‑19 pandemic period, which revealed that alcohol abuse/dependence was common among Chinese medical undergraduates. We also found that male medical undergraduates were more likely to report alcohol abuse/dependence than female students. Age and excessive daytime sleepiness were associated with alcohol abuse/dependence only among male students, whereas learning burnout and having a partner were associated with alcohol abuse/dependence only among female students. The above findings indicated the need for gender-specific screening and interventions for medical undergraduates to prevent alcohol abuse/dependence and its adverse consequences.

### The prevalence of alcohol abuse/dependence among Chinese medical undergraduates during the post-COVID‑19 pandemic period

Approximately 10.3% of the medical undergraduates reported alcohol abuse/dependence, slightly higher than of previously reported in China (4.57%-8.25%) [9, 10]. Some possible reasons may contribute to these differences. On the one hand, the difference might have been partially attributed to the different methods used to assess alcohol abuse/dependence. The two previous studies used the Alcohol Use Disorders Identification Test (AUDIT) to detect alcohol abuse/dependence, whereas our study used the AUDIT-C. On the other hand, the two previous studies were conducted before the COVID-19 outbreak, while our investigation was conducted after the outbreak of this pandemic. Previous studies suggested the prevalence of alcohol-related illnesses in the general population has increased after the pandemic outbreak [62, 63]. One of the possible reasons for this phenomenon is the subjective stress and mental distress caused by the COVID-19 pandemic, which was associated with the increase of alcohol consumption [64, 65]. However, to our knowledge, the present study is the first to investigate alcohol abuse/dependence among Chinese medical students after the outbreak of the COVID-19. Therefore, further research is needed to determine whether pandemics may have a negative impact on alcohol use and related disorders among medical students. Finally, although only 10.3% of the medical students in this study reported alcohol abuse/dependence, Chinese undergraduate medical enrolment in 2020 is already over 2 million [66], which means that a large number of students are potential victims of alcohol abuse/dependence. Therefore, timely screening for alcohol use and prevention of alcohol abuse/dependence is warranted, especially after the outbreak of the pandemic.

### Male medical students were at higher risk of alcohol abuse/dependence than female medical students

The present study demonstrated that the prevalence of alcohol abuse/dependence is significantly higher in males than in females, which was similar to the findings reported in other countries [5, 67]. The gender differences in the prevalence of alcohol abuse/dependence may be driven by biological and socio-cultural (sex-related) factors. On the one hand, previous studies have found that men release more dopamine than women when exposed to similar levels of alcohol, which is strongly associated with alcohol abuse/addiction [68, 69]. On the other hand, traditional gender roles have led to a tolerant attitude towards male alcohol consumption, i.e., heavy alcohol consumption by men is often viewed as a higher level of masculinity but alcohol use by women is regarded as indiscretion [70–72]. Although emerging evidence have shown that gender differences in problematic alcohol use have narrowed in the general population as gender roles have become more equal [24], our study still showed that male medical undergraduates are nearly 3 times more likely to engage in alcohol abuse/dependence than their female counterparts. Therefore, it is important to prospectively track the prevalence of alcohol abuse/dependence in different genders.

### Age and excessive daytime sleepiness were associated with alcohol abuse/dependence only among male students

A better understanding of gender-specific risk factors for alcohol abuse/dependence helps promote the wellbeing of medical students. Our findings suggest that older age and excessive daytime sleepiness were strongly associated with alcohol abuse/dependence in male medical students. Some other studies also suggested that the frequency and amount of alcohol consumption increased with age [38, 73, 74]. The possible reason for this is that as they get older, females and males are under increasing pressure to conform to culturally sanctioned gender roles [75]. In China, traditional gender roles tend to discourage or restrict female drinking, but accept and even expect male drinking [71]. Therefore, males may consume more alcohol as they age, thereby increasing the risk of alcohol abuse/dependence, suggesting that more attention needs to be paid to alcohol abuse/dependence among older medical students, particularly male students. In addition, after controlling for demographic variables, only excessive daytime sleepiness was independently associated with alcohol abuse/dependence among male medical students but not their female counterparts in our study. One possible reason for this is that men tend to self-medicate with alcohol more often than women when they have sleep-related problems [76]. Both this study and some previous studies have shown that over one third of medical students have experienced excessive daytime sleepiness [77–79]. Therefore, excessive daytime sleepiness among medical undergraduates needs more attention, and the improvement of sleep quality may help them reduce their problematic alcohol use, especially for male students.

### learning burnout and having a partner were associated with alcohol abuse/dependence only among female students

The present study also showed that having a partner and learning burnout were related to alcohol abuse/dependence among female medical students. Similarly, previous study indicated that alcohol abuse/dependence was more likely to occur in physicians who had a partner [18]. However, this association was not found among male medical students in our study. Previous studies suggested that female surgeons were more vulnerable to work-family conflicts, which appeared to be associated with their depression and alcohol abuse [80]. Female medical students with a partner might also be more vulnerable to the imbalance between study and relationship, which might be the reason for their alcohol abuse/dependence. Furthermore, our study found that only female medical undergraduates showed strong association between alcohol abuse/dependence and learning burnout. This was consistent with findings of other studies, which showed a significant association between the habit of drinking once or twice a week and burnout in female medical students only [81]. Notably, although a significantly higher prevalence of learning burnout was observed in male rather than female medical undergraduates, which was consistent with other studies [81–83], no significant association was found between alcohol abuse/dependence and learning burnout among male students. This might be due to the gender difference in motivations for drinking, e.g., women were more likely to drink due to coping issues while men were more likely to drink for social and recreational reasons [84, 85]. Thus, female medical students may be more likely to use alcohol as a coping strategy for learning burnout. Based on our findings, reducing learning burnout and improving the ability to cope with unpleasant emotions might be helpful to address the issue of alcohol abuse/dependence among medical students, especially for females. As we did not directly assess the motivations of alcohol drinking, we could only speculate that motivations of alcohol drinking might be the source of gender differences in alcohol abuse/dependence and deteriorating mental health. Nevertheless, the causality of this association can only be determined by further longitudinal studies.

### limitations of the study

There are several limitations to our study. First, this is a cross-sectional survey, which has limited the elucidation of the causality. Thus, future longitudinal studies are warranted. Second, this survey used convenience sampling and all the data were self-reported, which might have led to report bias; however, this might have been mitigated by the anonymity of the participants, as studies have shown that data from responders can be valid and reliable when their privacy is protected [86]. Third, this study lacked comparable pre-pandemic alcohol abuse/dependence among medical students to make direct comparisons. Finally, we used self-reported scales instead of standardized diagnostic tools to measure alcohol abuse/dependence and other mental illness, which may have created a potential bias that needs to be improved in future studies.

## conclusion

The present study showed that alcohol abuse/dependence was common among medical students during the post-COVID‑19 pandemic period. Substantial gender differences in the prevalence and risk factors of alcohol abuse/dependence among medical undergraduates were found in our study, which highlighted the importance of gender-specific screening and interventions for medical students to prevent problematic alcohol use and its adverse consequences. However, this cross-sectional study cannot explain the causal relationship between alcohol abuse/dependence and risk factors between female and male medical students, which need be confirmed by prospective cohort studies in the future.

## Data Availability

The data that support the findings of this study are available on request from the corresponding author. The data are not publicly available due to privacy or ethical restrictions.

## Abbreviations

COVID-19: The coronavirus disease 2019
AUDIT-C: The Alcohol Use Disorders Identification Test-Consumption
LBUS: The Learning Burnout of Undergraduates Scale
PHQ-9: The Chinese version of the 9-item Patient Health Questionnaire
GAD-7: The 7-item Generalized Anxiety Disorder Scale
ESS: The Epworth Sleepiness Scale
OR: Odds ratio
95%CI: 95% Confidence interval
CNY: Chinese yuan
